# Resilient critical infrastructures: An innovative methodological perspective for critical infrastructure (CI) integrated assessment models by inducing digital technologies during multi-hazard incidents

**DOI:** 10.1016/j.mex.2024.102561

**Published:** 2024-01-09

**Authors:** Ahmad Mohamad El‐Maissi, Moustafa Moufid Kassem, Fadzli Mohamed Nazri

**Affiliations:** a School of Civil Engineering, Engineering Campus, Universiti Sains Malaysia, Penang 14300, Malaysia; b AL Marshad Contracting & Precast Research and Development Department, Riyadh 12361, Kingdom of Saudi Arabia

**Keywords:** Geographic information system (GIS), Map models, Multi-hazard simulation programs resilient infrastructure, Emergency strategical platforms, Critical Infrastructure management, Gaming applications and scenarios, Virtual reality (VR) visualization tools, *Resilient Critical Infrastructures: An Innovative Methodological Perspective for CI Integrated Assessment Models by Inducing Digital Technologies during Multi-Hazard Incidents*

## Abstract

Over the last decade, the notion of community resilience, which encompasses planning for, opposing, absorbing, and quickly recovering from disruptive occurrences, has gained momentum across the world. Critical Infrastructures (CI) are seen as critical to attaining success in today's densely populated countries. Such infrastructures must be robust in the face of multi-hazard catastrophes by implementing appropriate disaster management and recovery plans. Given these facts, it is critical to establish a new methodological perspective with an integrated system for effective disaster management of CI, as well as an intelligent application that will aid in the construction of more resilient and sustainable cities and communities. This perspective proposes a holistic gaming scenario application for assessing the vulnerability and accessibility of critical infrastructures during multi-hazard events, with a primary focus on conducting an integrated assessment for critical infrastructures and their assets. Mainly, the perspective includes a holistic gaming scenario application that will aid in accurately quantifying geographical spatial information and integrating big data into predictive and prescriptive management tools using virtual reality.

• Conducting Integrated Assessment Models for evaluating vulnerability of Critical Infrastructures.
• Inducing Digital Technologies during Multi-Hazard Incidents for improving Natural hazard assessment models.
• Developing an open-world gaming scenario that is considered with high visual motion pictures and scenes.

## Graphical abstract

**Figure:**
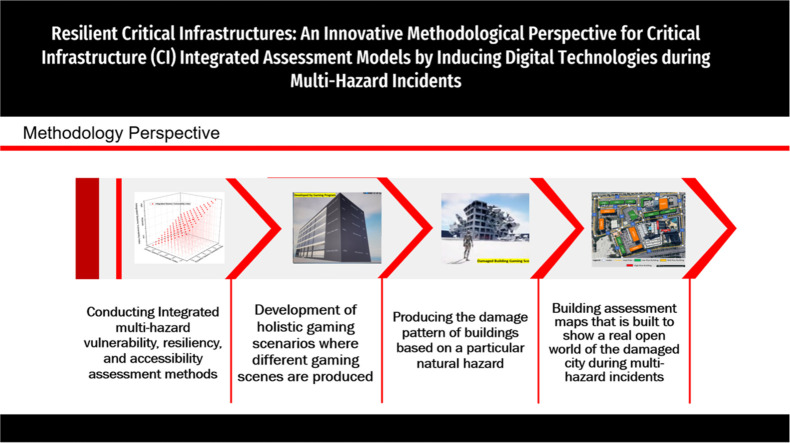

Specifications table

**Table:** 

Subject area:	Engineering
More specific subject area:	*Integrating Multi-hazard Models*
Name of your method:	*Resilient Critical Infrastructures: An Innovative Methodological Perspective for CI Integrated Assessment Models by Inducing Digital Technologies during Multi-Hazard Incidents*
Name and reference of original method:	El‐Maissi, A. M., Argyroudis, S. A., Kassem, M. M. and Nazri, F. M. (2022) 'Integrated Seismic Vulnerability Assessment of Road Network in Complex Built Environment toward more Resilient Cities', Sustainable Cities and Society, pp. 104363.
Resource availability:	*Not Applicable*

## Method details

 

## Background

The proper operation of Critical Infrastructures (CI) during natural disaster situations is considered to be critical for protecting the safety, social, and economic prosperity of various communities across the world. An infrastructure is designated critical if its destruction or disruption has a major impact on safety, security, and social well-being [1], [2], [3] . However, these CI cannot be treated as isolated infrastructural systems, but instead, the correlated chain between them should be examined in order to establish an efficient integrated CI system. The malfunction of one CI can cause a cascading failure for multiple infrastructures with possible disastrous implications on integrated infrastructural system [4]. Moreover, there are two types of damage that could influence critical infrastructure. The first type is intrinsic or direct damage that is caused directly from different types of hazardous incidents on the triggered system itself i.e., structural and geotechnical failure of piers in bridges, columns failure in buildings, and damaged embankments for roadway systems. On the other hand, the second type is eccentric or indirect damage that is described by the cascading effect of CI on other surrounding one in integrated infrastructural system such as, the affect of collapsed building debris on the surrounding road networks or the cascading effect of road embankments on different lifelines beneath roadway systems [5].

**Fig. 1:**
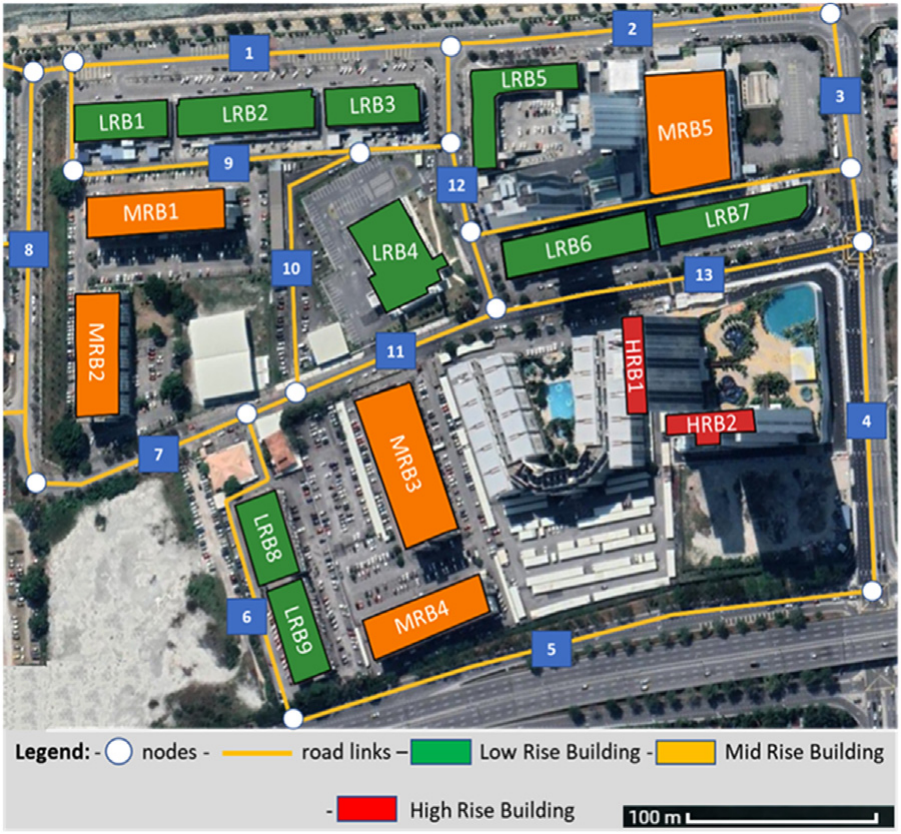
Sample of investigated elements in integrated vulnerability assessment approach in Penang Malaysia.

Due to these facts, the integration between intrinsic and eccentric characteristics for CI is considered vital in vulnerability assessment. Nevertheless, assessing the interdependency between different CI is an important factor that should be considered when assessing the vulnerability of integrated infrastructural system. Fig. 1 represents a sample of different investigated elements in Penang, Malaysia, which are the road network and the buildings that are surrounding this network. The elements vulnerability is investigated for roads and buildings separately in the first step. This is followed by finding the cascading effect of buildings on roads disruption or damaged roads on the accessibility to critical buildings such as hospitals, airports, firefighting station, power stations, and governmental buildings. In Fig. 1 In Fig. 1, the buildings are categorized to Low Rise Building (LRB), Mid Rise Building (MRB), High Rise Building (HRB) based on their geometrical aspects. Moreover, the road network is divided based on the building's setback distance from the main road.

This research is not focusing on the technical development of the integrated vulnerability assessment method, where, it has been developed and investigated in our previous research studies [5,6]. Mainly, the focus in this studies on the challenges facing the development of the CI integrated assessment models during multi-hazard incidents, the importance of deploying the integrated vulnerability assessment models and indices for advancing the disaster risk reduction strategies, and the role of digital technologies in improving these integrated assessment models.

## Challenges in the development of the CI integrated assessment models

Unfortunately, the developed applications are restricting the assessment procedural method, where the researchers are using different models and applications to investigate various factors and implications and sometimes they are using manual procedures that are very complicated and time-consuming. For example, when investigating earthquake vulnerability specific applications are used to develop fragility functions or resiliency functions. However, different GIS applications are used to assess the accessibility of roads during earthquake incidents, and the calculation of the Vulnerability Index (VI), Resiliency Index (RI), and Accessibility Index (AI) is still done using manual methods. Still, all of these procedures are done without including the effect of another sequenced natural hazard, where the inclusion of other incidents needs another application leading to unprecise and nonsequential results.

Nevertheless, the visualization of the main results is still considered primitive in the developed applications, where the applications can not reflect the results directly through maps or functional curves. The previous applications only give the calculated value of the assessed rates by which, the user of this application should put tremendous effort to develop the functionality curves and build the visualization maps through different applications by including the values one by one and building the whole network, structural, and infrastructural systems.

Moreover, nowadays gaming software and applications are being used to build open-world games that are considered with high visual motion pictures and scenes. However, these vital gaming tools are not being efficiently used in the management and assessment of CI during multi-hazard incidents where the gaming applications can reflect the impacts of natural hazards with great visual maps and scenes. Additionally, Virtual Reality (VR) has made a technological revolution when considering the interaction with the open world, but, this interesting technology is not being used in the previous developed applications. Mainly, VR can be considered impactful in interacting directly with these types of disastrous incidents, where the real open world during disasters can be reflected directly in motion pictures. Mainly, a holistic gaming scenario for multi-hazard assessment is an approach that considers various hazards and their potential impacts on a system or environment. The holistic aspect of this gaming scenario involves considering the interconnectedness of different hazards and their potential cascading effects.

The present research has been produced to highlight these issues as well as other factors to integrate the different assessment factors and the interdependencies between various CI under the effect of multi-hazard incidents, by developing a holistic method with the inclusion of innovative technologies for better visualization aspects and more precise and sequential results that can be managed through governmental and organizational applications

## Multi-hazard incidents and their effect on the sequenced damage on critical infrastructure (CI)

Multi-hazard incidents can cause a catastrophic situation for any society, even for countries that have good mitigation measures and preparedness it is considered challengeable to face this type of incidents. A single hazard such as, earthquake, tsunami, landsides, and floods can have a linking sequence where one incident can trigger another, or they can happen simultaneously at the same time. Due to this reason the consequences are very dangerous and the emergency evacuation plans can breakdown. Mainly multi-hazard incidents can be classified into different categories (Independent hazards of different impacts, correlated or cascading hazards, correlated or independent hazards of the same nature) and can be linked with different terminologies (Interaction, trigger, dependence, combination, cascade, domino and other terminologies) as described by [7,8]. The main aim of this research is to focus on the sequenced damage that can result from this type of incidents and what are the main challenges that face the researchers and emergency planners in containing them. For instance,

## Importance of integrated vulnerability index model

Most of the of the developed applications do not take into consideration the integration of these factors and their impact on each other or the impact of various natural hazards at the same time, whereas most of the developed algorithmic models focus on single structural or infrastructural systems without considering the holistic integrational interdependencies between these systems.

Due to these facts and based on our previous research that have been developed for Penang state in Malaysia as an integrated seismic approach to assess the Intrinsic and Eccentric Vulnerability Index (ISVI and ESVI) during earthquake incidents, where the ISVI focus on the external characteristics of the system itself, while the ESVI studies the effect of external factors of one infrastructure on another. Hence, its clear that the integrated approach is considered more efficient when compared to single assessment models that focus on one criterion without considering the full holistic approach. For more information about ISVI and ESVI methods check El-Maissi et al. [6].

**Fig. 2:**
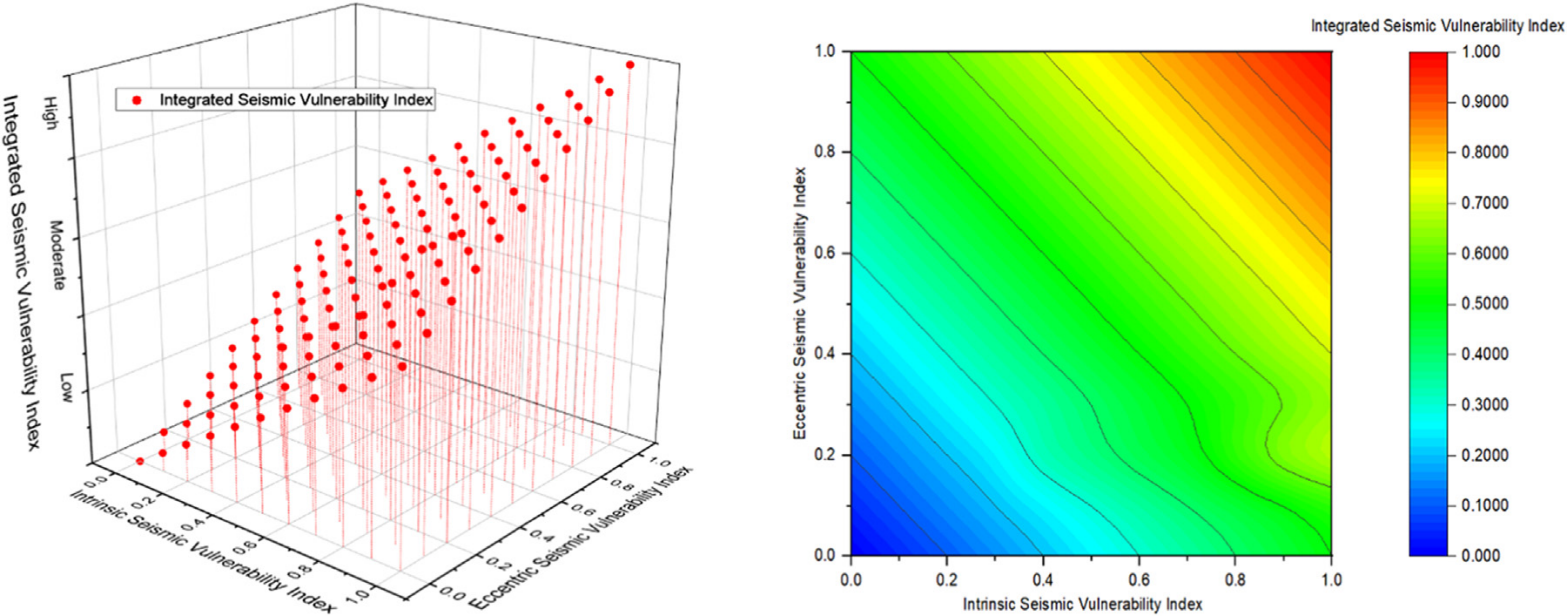
integrated seismic vulnerability assessment values based on previous developed models reproduced from [6].

Fig. 2 represents the main results that are concluded form the integrated seismic approach, where as shown in Fig. 2 when considering the ISVI or ESVI solely at extensive stage which is equal to 1 the results are showing moderate vulnerability index. However, when both indices are considered together the vulnerability index is increasing to high state. Hence, the integrated perspective is considered more effective by 50 % to assess the damage that can happen to different Critical Infrastructure (CI) during earthquakes or any other natural hazard incident.

## Main obstacles of traditional assessment models that investigates natural multi-hazard incidents

The inclusion of digital technologies in the assessment of multi-hazard incidents is considered an important step for the improvement of the Critical Infrastructure (CI) integrated assessment models. Unfortunately, the produced software applications limit the assessment procedural approach, in which researchers use multiple models and applications to explore various elements and consequences, and occasionally they use manual methods that are highly difficult and time-consuming. When researching earthquake vulnerability, for example, specialized applications are employed to generate fragility functions or resiliency functions. However, multiple maps that are extracted from Geographic Information System (GIS) software are used to analyze road accessibility after seismic disasters. Moreover, The Vulnerability Index (VI) that measures the susceptibility and exposure of a system or population to hazards, the Resiliency Index (RI) which, quantifies the capacity to recover and adapt after experiencing hazards, and the Accessibility Index (AI) that works on evaluating the ease of accessing essential resources and services within a given area are still calculated manually. Nonetheless, all of these methods are carried out without considering the influence of another sequenced natural hazard, where the incorporation of further occurrences necessitates another application, resulting in imprecise and nonsequential outcomes. Nonetheless, the depiction of the key outcomes in produced applications is still regarded rudimentary, as the applications cannot directly represent the results through maps or functional curves. The previous applications only provide the calculated value of the assessed rates; therefore, the user of this application must expend considerable effort to produce the functionality curves and build the visualization maps through various applications through including the results one by one and building the entire network, structural, and infrastructural systems.

Furthermore, gaming tools and applications are currently being utilized to create open-world games with high visual motion images and scenarios. However, these critical gaming technologies are not being used effectively in the management and assessment of CI during multi-hazard situations, where gaming applications may portray the consequences of natural disasters with excellent visual maps and sceneries.

The present research is introduced in response to these issues as well as other factors to integrate the different assessment factors and the interdependencies between various CI under the effect of multi-hazard incidents, by developing a methodology to build a holistic seismic vulnerability assessment technological application for better visualization aspects and more precise and sequential results that can be managed through governmental and organizational mobile version websites and different public web portals.

## An innovative methodological perspective for CI integrated assessment models by inducing digital technologies during multi-hazard incidents

This research is introducing an innovative methodological perspective for CI integrated assessment models by inducing digital technologies during multi-hazard incidents. It is a methodology that integrates multiple multi-hazard assessment criteria for various Critical Infrastructure (CI), mobile apps, interactive Geographic Information System (GIS) maps based on the hazardous scenario, and Virtual Reality interfaces (VR). During a multi-hazard occurrence, the integration operates through a single application on computing devices that is built based on this methodological perspective to examine the interdependencies, vulnerability, resilience, and accessibility characteristics.

**Fig. 3:**
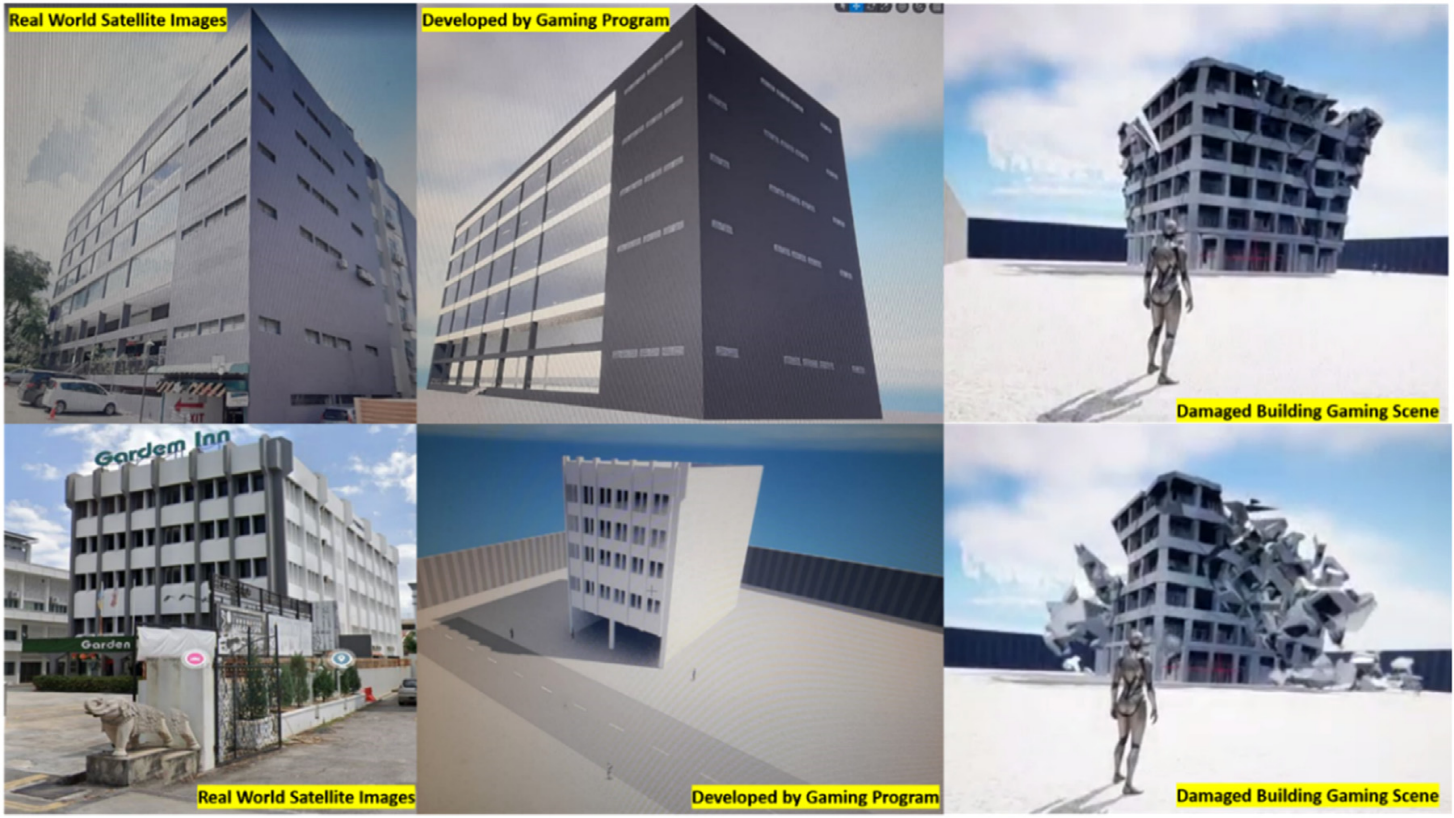
Schematic program outcome of holistic gaming scenario application of critical infrastructures during multi-hazard incidents.

Fig. 3 illustrates a sample of buildings created by a gaming software (Unreal Engine [9]) to generate the damage pattern of buildings based on a particular natural hazard, with the figure displaying the conceptual program outcome of holistic gaming scenario application of CI during multi-hazard incidents.

**Fig. 4:**
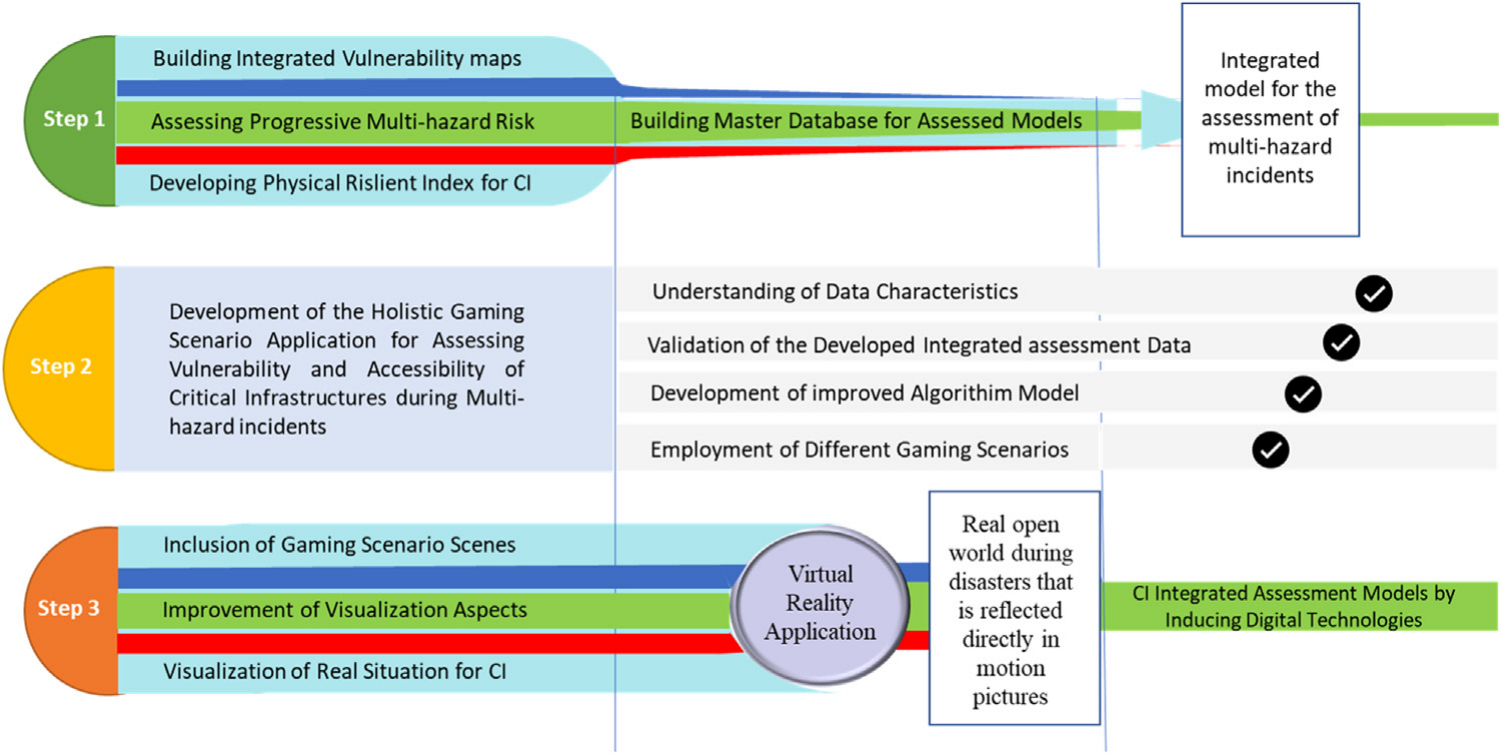
A methodological perspective for development of CI integrated assessment model during multi-hazard incidents that is induced with innovative digital technologies.

Nevertheless, Fig. 4 represents the main methodological perspective that is developed to employ the CI integrated assessment models through including the digital technologies during multi-hazard incidents, where the method is divided into three main steps. In step 1 the integrated model for assessment of multi-hazard incidents is developed through building a master of the developed assessment models which are, the construction of integrated vulnerability maps using the assessed Vulnerability Index (VI) for different natural hazard incidents, assessment of progressive multi-hazard risks that is resulting form the sequenced effect of one natural hazard on another (i.e. seismic activity that triggers tsunami incidents), and development of Resilient Index (RI) that is conducted by employing of direct loss of resilience (LOR) and development of resiliency and functionality curves.

This is followed by step 2 that focuses on the development of holistic gaming scenarios where different gaming scenes is produced through understanding the data characteristics, validation of the extracted assessment data, development of improved algorithm model through using gaming platforms such as Unreal Engine, and at last stage different gaming scenarios are employed to reflect the main damaged infrastructure at different damage states.

Subsequently, a virtual reality application is built to show a real open world of the damaged city during multi-hazard incidents and this is mainly reflected through inclusion of the gaming scenario sciences at different areas of the city and for different Critical Infrastructure (CI) and improvement of the visualization aspects by improving the resolutions for more realistic sciences that can be used by emergency aid facilities to visualize the real evacuation procedures that can be done during multi-hazard incidents.

## Concluding remarks

The represented innovative methodology is regarded as critical for establishing techniques that can reduce fatalities and injuries during multi-hazard occurrences. The current innovation is regarded detectable for Critical Infrastructures (CI) and their assets, where they can recover quicker and operate more effectively following a series of hazardous events, in order to appropriately reactivate the economic and social cycle. Furthermore, the data collected by the models will help in rescue attempts and other emergency activities in the affected region. This project will help to build a resilient infrastructure as well as a safe and sustainable living environment. This work confirms the high importance of applied sciences in different fields as shown in many scientific papers published before [10,11].

Subsequently, the findings of this innovation will have a considerable impact on the evolution of multi-hazard seismic assessment methodologies, notably in the domains of tourism, disaster risk management, environmental research, and development. It may also make a significant contribution to society in terms of awareness, readiness, and the economic and social cycle.

## Declaration of competing interest

The authors declare that they have no known competing financial interests or personal relationships that could have appeared to influence the work reported in this paper.

## Data Availability

No data was used for the research described in the article.
